# Gleanings

**Published:** 1896-06

**Authors:** 


### GLEANINGS.

A threatened reopening of an old case of fistulous withers was successfully averted by large doses of salicylate of soda and iodide of potash, given in hot bran-mashes, with a denial of all grain. Externally the use of Dick's white lotion applied three times daily. The use of strong caustics, escharotics, or other destructive agents in fresh cases of fistulous withers often does a great deal of harm and destroys a large amount of tissue which requires a long time to restore. Cleanliness and dry dressings, with the openings well covered with a pad of oakum and a clean cloth, will ofttimes insure a prompt and thorough recovery.

Paralysis of the glottis was recently met in a bitch two weeks after whelping. Death ensuing, a post-mortem revealed a complete breaking-down of the uterus and horns, and either the absorption of septic matter or reflex disturbance was looked upon as the causative factor of the paralysis. One of the puppies presented a septic condition of several of the toes, with a deep sloughing of the parts affected, which was the only noticeable abnormal condition of mother or offspring from the time of whelping to the development of the loss of power in deglutition of the mother.

The Department of Agriculture is out in a circular (No. 8) calling the attention of our stockraisers and producers of animal food-products to the great market that centres at Manchester, England. An interesting description of the completion by the Manchester corporation of one of the largest plants for the reception of live animals, their suitable maintenance for a period, the most improved abattoirs, and cold-storage facilities of unsurpassed completeness, all located adjacent to the great Manchester ship-canal, and opening up to trade a field of consumers numbering 7,500,000 people.

All about the Dog. Seventeen species of wild dogs are known. The most famous dog-artist was Landseer. The native wild dog of Australia is called dingo. The Laplanders called the bear "the dog of God." The "dogs of war" are famine, sword, and fire. The mastiff is believed to be indigenous to Thibet. A dog is fully grown at the end of his second year. The dog is mentioned thirty-three times in the Bible. Cerebus was the famous "three-headed dog of hell." The temperature of the dog's blood is 102° Fahrenheit. Irish and Danish hounds are the largest dogs of Europe. The lycacon of the Cape of Good Hope is a native mastiff. In 1876 there were licensed in Great Britain 1,362,176 dogs. The dogs of Orion were named Arctophomus and Ptophagus. In Siberia the Russian greyhound is used for tracking fugitives. The greyhound appears on the oldest Egyptian monuments. In Ireland there are four packs of stag-hounds with 100 couples. The famous Cuban bloodhounds are descendants of the mastiff. Only domesticated dogs bark; in the wild state they howl or whine. The pariah curs of India are the direct descendants of wild dogs. The fossil remains of four different types of dogs have been found. There are 20,000 hounds in Great Britain used for hunting purposes. The Esquimaux dog is found in Siberia, as well as in North America. All Arctic dogs are provided with a thick mat of wool under their hair. There are over 600 proverbs in the English language relating to dogs. Shepherd dogs used in caring for sheep are not taxed in Great Britain. Diogenes was called "the dog" on account of his habitual crustiness. The mastiff was known to the Greeks in the time of Alexander the Great. The pupil of the dog's eye, like that of the other diurnal carnivora, is round. Mulhall computes that there are at present 2,000,000 dogs in Great Britain.—*St. Louis Globe-Democrat.* A Newfoundland dog prevented an eagle from carrying away the baby of William Sloane, in Knott County, Ky.—*Philadelphia Evening Bulletin.*

Frank Shaw, President of the New York State Tuberculosis Commission, is authority for the statement that no tuberculosis exists among the cattle of the Isle of Jersey, and that the records of the Commission's work show that there are more tuberculous cattle in other breeds than in Jerseys.

In very young dogs and those cases where they have become anæmic from faulty feeding, and where one suspects the existence of worms, the syrup of iodide of iron is a useful mixture, given in fifteen to thirty minim doses. Where diarrhœa exists the addition of paragoric aids in correcting the trouble.

